# Impact of an Online Parenting Support Programme on Children’s Quality of Life

**DOI:** 10.3390/children9020173

**Published:** 2022-01-31

**Authors:** Cristina Nunes, Cátia Martins, Marta Brás, Cláudia Carmo, Andrea Gonçalves, António Pina

**Affiliations:** 1 Psychology Research Centre (CIP), Universidade do Algarve, Campus de Gambelas, 8005-139 Faro, Portugal; csmartins@ualg.pt (C.M.); mbras@ualg.pt (M.B.); cgcarmo@ualg.pt (C.C.); 2 Departamento de Ciências Sociais e da Educação, Universidade do Algarve, Campus de Gambelas, 8005-139 Faro, Portugal; amdesousagoncalves@gmail.com; 3 Administração Regional de Saúde do Algarve, 8005-145 Faro, Portugal; apina@arsalgarve.min-saude.pt

**Keywords:** children, health promotion, online parenting programme, parents, positive parenting, quality of life

## Abstract

The study aims to describe the parental use of an online parenting support programme, the ‘Open Window to Family’ (JAF) and to evaluate its impact on perceived children’s quality of life (QoL). This programme makes online resources available to support positive parenting. The study included 363 parents (*n*_intervention group_ = 142) who completed measures to evaluate their children’s QoL. The results suggest that using the programme for a longer time and accessing more information/services are positively related to the perception of utility but not to the frequency of use. The programme proved to be more useful for specific difficulties and to search for specific information. We found high levels of parental perception of children’s well-being, both physical and psychological, and lower values in social support and relationships with peers. No differences were observed between the total QoL of children in the intervention group and control group. The differences in the dimensions of QoL are due to interaction with the level of education of the mother: mothers with higher education reported higher physical well-being, social support, relationships with peers, and school context. Guidelines are suggested to enhance the accessibility of this type of programme as well as enhance its impact on parents and children.

## 1. Introduction

In recent years, research in the family field has highlighted the importance of parenting training and support programmes as well as the evaluation of these programmes. These programmes provide an individual development process that allows parents to enhance their abilities to feel, imagine, understand, and use knowledge to parent [1].

There are many studies that show that these programmes reduce family stress, improve parent–child relationships, increase child well-being and may be effective in preventing maltreatment [2]. This effectiveness depends on the appropriateness of the programme to the needs and characteristics of the families, as well as on meeting quality criteria [3,4,5]. However, the number of evidence-based parenting support programmes in Portugal is still small, and evaluations of their effectiveness are scarce [6,7].

Family support policies were significantly reinforced with the publication of Recommendation 19/2006 of the Council of Ministers of the European Union on positive parenting [8]. This recommendation contributed to the consolidation of measures to prevent child abuse and promoted universal access to programmes to promote parenting skills, namely, through online support [9]. Several online parenting support programmes have been developed since the 1990s. Studies have shown that parents perceive the Internet as a potentially useful and accessible support tool in educating their children and promoting family and child well-being [9,10,11]. The extension of its use into the educational sphere was thus an expected development. Muñetón et al. [12] observed that the topics related to children’s education that were most frequently searched online by parents were: school (40%), family health (40%), parenting advice (36%), behaviour problems (28%), and psychological development (26%). 

Online parenting promotion programmes that follow international quality guidelines by promoting contact with professionals and other families allow parents to learn and exchange experiences about parenting and thereby increase their levels of empowerment [13]. However, some authors have warned about differences in the use of these resources (i.e., digital inequality) [14] due to circumstances such as parents’ sociodemographic conditions and geographic location [15]. Indeed, socioeconomic status (SES) has been found to be a critical factor in quality of life and family functioning [10].

This is a current concern of health professionals and education and community agents who intervene in the promotion of health and quality of life [16]. With these concerns, health professionals in Portugal, more specifically, in Algarve, created the ‘Open Window to Family’ programme (JAF) [17], which consists of providing future and current parents with information in different formats (e.g., practical guides, videos) through a system of free access by means of an online page. This project began in 2007 as a partnership between the Algarve Regional Health Administration (ARS) and the two units of Algarve Hospital and University Centre (CHUA). It consists of a platform with resources to support the exercise of conscious parenting (i.e., increase awareness of yourself in parenting) and to promote harmonious development and access to professionals (e.g., doctors, psychologists, and others) with the help of digital technologies in order to clarify doubts and provide information directed to pregnant and parenting women, which covers the stages of development (from pregnancy to adolescence) and the most frequent and relevant health problems. Registration is completed online by answering a questionnaire that contemplates the collection of information (e.g., contacts, children, and other data).

The structure of the JAF platform includes several types of information and resources, namely: (1) ‘Programme and team’ (e.g., presentation, contact forms and news); (2) ‘Bulletins’ (17 booklets dedicated to developmental stages); (3) ‘Library’ (resources related to babies, children, adolescents, family and institutions, some translated into different languages); (4) ‘Pedagogical videos’, specially developed and covering 11 different thematic areas (e.g., pregnancy, safety, health problems); (5) ‘Useful links’ organised in three major areas—maternal health, child health, and parenting; and (6) ‘FAQs’, structured in themes that answer 90 questions. In addition to providing access to resources, the JAF platform allows for the elaboration of messages, which can be sent directly or by other available means.

Previous evaluations of family satisfaction with JAF suggest a gradual increase in participation (from 19 responses received in 2008 to 72 in 2013), access and frequency of use of online resources. The level of satisfaction with the programme has been in the upper-middle range and increased between 2008 and 2011, with the percentage of participants feeling very satisfied rising from 16% to 47% [17].

Most parenting support programmes are face-to-face; although in recent years, there has been an investment in online programs. Given their innovative nature, these programs still lack regular evaluations of their effectiveness by different stakeholders. As these types of interventions are aimed at parents and have as their primary goal improving the quality of life of children, evaluations of the effectiveness of these programs, including the JAF, have focused on parents as direct participants (e.g., satisfaction, knowledge, and parenting skills), thus justifying the study of the impact of these programmes on children.

The region where the program was developed is the southernmost region of Portugal. This region has a high number of families coming from other regions of the country, thus making it difficult to obtain extended family and social support. The online access to health professionals and the sharing of experiences with other families intends to be an added value for these families. A better understanding of the impact of online parenting support programs, may contribute to the improvement of online resources and increase positive parenting.

Although Spencer et al. [11] pointed to online parenting programs having a strong effect on the promotion of positive parenting and parents’ reinforcement [11], a more accurate assessment is needed, mainly concerning effectiveness analysis and the impact on children’s outcomes [11,18].

This study aims to describe the parental use of the JAF programme and to assess its impact on the perceived quality of life (QoL) of their children. 

## 2. Materials and Methods

### 2.1. Design

This is a retrospective analytical study with a control group, which compares a sample of parents participating in the JAF programme (intervention group—IG) with another sample of parents who did not receive this intervention (control group—CG).

### 2.2. Participants 

Participants were 363 parents (96.15% mothers) between 23 and 57 years old (*M* = 39.55, *SD* = 5.40). The IG involved 142 parents and the CG involved 221 parents (control/case ratio = 1.5). The inclusion criteria to IG were parents who had participated in the JAF programme and had children born between 1 January 2007 and 31 December 2010. To CG, the inclusion criteria was parents who had children with the same age of those from IG and had not participated in the JAF programme. 

### 2.3. Measures

#### 2.3.1. Sociodemographic Data

We used an ad hoc questionnaire to gather information about parents’ gender, age, level of education, and occupation. We also gathered information about children’s gender, age, schooling, failures, physical and mental health problems, number of siblings, and type of family.

#### 2.3.2. Process Outcomes of JAF 

We collected information about the length of use of the programme (in years), the use of programme information and services (from 1 = almost never to 4 = many times), frequency of use (1 = occasionally, 2 = monthly, 3 = biweekly, 4 = weekly; 5 = daily), programme usefulness (from 1 = not at all useful to 4 = very useful), and participation in other parental programme training (No/Yes).

#### 2.3.3. Child Quality of Life 

We used the KIDSCREEN-27 questionnaire [19]. It evaluates the perceived QoL of children and adolescents between 8 and 18 years old, and it was developed simultaneously in representative samples of 13 European countries. It can be used for screening, monitoring, and evaluation in national and international European health surveys. The Portuguese version was validated by Gaspar and Matos [19]. It includes 27 items that assess five dimensions of the child’s subjective well-being and health: (1) physical well-being (e.g., ‘Did your child feel good and fit?’); (2) psychological well-being (e.g., ‘Was your child in a good mood?’); (3) autonomy and family (e.g., ‘Was your child able to talk to you when he wanted to?’); (4) peers and social support (e.g., ‘Do you think your child was able to trust friends?’); and (5) school environment (e.g., ‘Was your child a good student at school?’). The response scale consisted of five options ranging from 1 = nothing to 5 = totally. Higher values correspond to a better perceived quality of life. We also calculated the general index that results from the sum of the items (Total QoL) and obtained good reliability coefficients: physical well-being α = 0.86; psychological well-being α = 0.79; autonomy and family α = 0.81; peers and social support α = 0.88; school environment α = 0.88.

### 2.4. Data Collection

The study was carried out in several steps. The first step consisted of the preparation and testing of the assessment instruments. The research protocol was prepared and tested with a group of parents specially selected for this purpose. Comments and suggestions from participants and the JAF team were examined and included as appropriate in the final version. The second step, online completion of the questionnaire by the IG, was then carried out. The questionnaire was made available online in 2019 and the JAF coordinator sent the link by email to all parents who had participated in the programme and had children born between 1 January 2007 and 31 December 2010 (*n* = 1353). To increase the response rate, the request for collaboration was repeated three times. In total, 149 responses were obtained. The third step consisted of the completion of the questionnaires by the CG: the research team contacted parents of the CG through after-school clubs in the Algarve where the JAF had not yet been implemented. Although it was not easy to implement control strategies for possible confounders, we try to collect parents for CG with homogeneous characteristics with IC. The completion of the questionnaires took place in the second half of 2019. Finally, in the fourth step, the parents of the CG had the opportunity for ethical reasons to participate free of charge in a training session on positive parenting.

All participants signed a written informed consent and were informed of the study’s aims, the non-compensatory nature of participation, the anonymous and confidential nature of their responses, and the possibility of withdrawing at any time without any negative consequences.

### 2.5. Data Analysis

The IBM SPSS–24 and MS Excel programmes were used for data results analysis and graphical representation. After checking the randomness of the data using the Little’s MCAR test, data quality was analysed and missing data were extrapolated using the SPSS missing value (EM algorithm). Questionnaires with more than 10% of items missing were excluded. Statistical assumptions for parametric analyses were checked in accordance with the recommendations of Tabachnick and Fidell [20]. 

After verifying the assumptions of normality, univariate ANOVA and factorial ANOVA were used to compare the results between groups. MANOVA was used to analyse multiple dependent variables. In the case of comparison of nominal variables, the Chi-Square test was used. Pearson correlations were used to study the relationships between scale variables. Results were considered significant if *p* ≤ 0.05, but *p* ≤ 0.10 was also reported. The effect size was calculated to clarify the degree of accuracy of the statistical judgments and the strength of the relationships between the variables.

## 3. Results

### 3.1. Participants’ Sociodemographic Characteristics

Most participants in both groups were mothers (97% in IG and 96% in CG). No significant differences were observed regarding gender participants between CG and IG (*χ*^2^ (1, 36) = 0.68; *p* = 0.410; *V* = 0.04). In the CG, parents were between 23 and 57 years old (*M* = 39.44; *SD* = 5.71) and those in the IG ranged between 24 and 50 years old *(M* = 39.71; *SD* = 5.89), with no significant differences observed between the groups (*F* (1, 363) = 0.22; *p* = 0.640; *ɳ*^2^ = 0.00). 

No differences were found significative (*χ*^2^ (1, 358) = 0.06, *p* = 0.810, *V* = 0.01) regarding the type of family (IG: single parent = 17%, biparental = 81%; CG: single parent = 18%, biparental = 81%). Parents from IG had more children (*M* = 2.18, *DP* = 0.68) than parents from CG (*M* = 1.76, *DP* = 0.83) but with a small size effect (*F* = 24.91; *p* = 0.000, *ɳ*^2^ = 0.06; see Table 1).

Participants’ children were on average 9.56 years old (*SD* = 1.35; range = 8–12), with no significant differences between groups (*F* (1, 357) = 0.66, *p* = 0.417, *ɳ*^2^= 0.00; see Table 1). As for gender, 57% were boys in CG and 55% in IG, also without any significant differences between the two groups (*χ*^2^ (1, 362) = 0.19, *p* = 0.661, *V* = 0.02). In respect to school withholdings, most of the children had success (IC = 95%, CG = 89%) and the differences between groups were small and not significant (*χ*^2^ (1, 226) = 2.69, *p* = 0.101, *V* = 0.11). Health problems were not frequent (IC = 4%, CG = 8%) and the differences were not significative and with a small effect (*χ*^2^ (1, 226) = 2.43, *p* = 0.119, *V* = 0.10). 

Regarding education level, 38% of all participants had completed secondary education, 34% had completed university education, and 28% had completed basic education. Parents in the IG had a significantly higher level of education (34% secondary education, 53% university education) than those in the CG (22% secondary education, 41% university education), with a moderate effect size (*χ*^2^ (2, 363) = 41.99; *p* = 0.000; *V* = 0.34). 

In the CG, most participants had professions with low (32%) and medium (42%) qualifications, while in the IG, qualifications were medium (42%) or high (54%). This difference was statistically significant with a moderate size effect (*χ*^2^ (2, 328) = 47.06, *p* = 0.000, *V* = 0.38).

### 3.2. Process Outcomes of the JAF Programme 

The IG’s parents had used the JAF programme for approximately 7 years (*M* = 7.38, *SD* = 3.72, amplitude = 0–14), and it was the only parenting support programme used by the vast majority (93.53%). Parents from CG had never attended a structured parenting support programme. The programme’s perceived usefulness was medium–high (61% considered it useful and 33% very useful).

Table 2 displays the correlation indices between the modalities of use and the perceived usefulness of the JAF. We observed that the parents who more often used the information and services provided by JAF were also the ones who considered the programme more useful and who used the programme longer and more often. Additionally, parents who had known the programme for the longest time were the ones who used it most often and who rated it as most useful. The frequency of use of the programme was not significant related with the perception of programme usefulness.

### 3.3. Children’ QoL

The children’s QoL levels reported by parents of both groups were high, namely, in psychological well-being (*M* = 4.30, *SD* = 0.49) and physical well-being (*M* = 4.22, *SD* = 0.75). All dimensions of QoL were positively and significantly related (Table 3).

Differences between IG and CG in the Total QoL of children were analysed using a factorial ANOVA, including Group as an independent variable (0 = CG; 1 = IG) and controlling for parents’ education level (0 = primary and secondary education; 1 = university education). This factorial ANOVA did not reveal significant differences between groups (*F* (1, 353) = 1.49; *p =* 0.223; *ɳ*^2^_partial_ = 0.004; see Table 4).

The MANOVA analysis including the QoL dimensions showed that the differences found between the groups (CG and IG) were due to the interaction of the parents’ educational level (*Λ* = 0.94; *F* (5, 346) = 4.42, *p* = 0.001, *ɳ*^2^_partial_ = 0.06; see Table 5). 

Subsequent ANOVAs showed that children of mothers with university studies had better QoL in physical well-being (*F* (1, 353) = 3.90, *p* = 0.049, *ɳ*^2^_partial_ = 0.01) and peers and social support (*F* (1, 353) = 4.73, *p* = 0.030, *ɳ*^2^_partial_ = 0.01), both with a small size effect, and in school environment (*F* (1, 353) = 17.11, *p* = 0.000, *ɳ*^2^_partial_ = 0.05) with a large size effect.

In IG, children age is related negatively with QoL (*r* = −0.22, *p* < 0.05), physical well-being (*r* = −0.27, *p* < 0.01), and autonomy and family (*r* = −0.18, *p* < 0.05), and positively with psychological well-being (*r* = 0.23, *p* > 0.01). Children gender correlates negatively with school environment (*r* = −0.18, *p* < 0.05). School withholding reveal a negative association with school environment (*r* = −0.22, *p* < 0.05).

We observed significant positive relations between the use of the information and services programme by parents and QoL. Significant positive relations were also found between children’s QoL and parents’ positive opinions about the usefulness of the programme (Table 6).

## 4. Discussion

Considering the potential role that online programmes can play in providing support for the exercise of positive parenting as well as in promoting child and family well-being [9], this study assessed the impact of parental use of the JAF programme on the perception of child QoL. To this end, a comparative and retrospective study was carried out between parents who have used the JAF programme and a control group composed of parents who have never participated in the programme. 

The first relevant result shows that the JAF programme may contribute to fill an unmet need to support parents in performing their rewarding but difficult task of parenting. For control purposes, no CG participant had previously attended any type of programme, including JAF, and only 6.47% of the intervention group had done so in the first evaluation moment.

Since a random distribution of exposure was unfeasible, as well as a matching strategy for confounders control, the design was necessarily observational and subject to limitations, mainly due to self-selection. We try to control it using statistical methods (i.e., MANOVA) to analyse possible confounders variables (e.g., parents’ educational level, age, sex, family type, and children). An analysis of the sociodemographic characteristics shows that although similar in many aspects, the IG had a higher educational level and more qualified professions, suggesting that higher social classes have greater propensity and capacity to benefit from these services; although, they are probably the group that needs it less. The disparity between low- and high-income families has been highlighted, not only regarding Internet access but also in various online activities designed for parents [10,21]. Hargittai [14] suggested that when analysing parental equity in online access to parenting support resources, one way of reducing the possibility of digital inequality is the integration of factors such as the social contexts of the Internet use and the diversity of people skills’ for using technologies.

These data are consistent with the user profiles for this type of programme. In a review study about profile users of online services for parents, Daneback and Plantin [22] found that 85% to 95% were middle-class mothers. Santos et al. [23] also highlighted the existence of significant differences between socioeconomic groups in Internet access, especially with regard to their children’s educational support, with parents who have higher educational levels tending to search for and benefit from more information. Several investigations have identified education as an important predictor of digital usage [10], constituting a relevant risk factor.

The great predominance of women in both groups of participating parents suggests that there might be an access barrier for men to the programme. It would be very useful to know more about this to better overcome it. We do not know if it is due to a lack of interest, perceived usefulness, propensity to ask for help and advice, or a poor adaptation of the program’s contents to the specific needs of parenthood. In fact, in this context, few health services have specific policies aimed at males; although, this need is emerging [24].

The gender distribution of children was also skewed but in the opposite direction. The proportion of boys (57%) was significantly higher than expected (51%), as it is known that the proportion of boys aged 5 to 14 years residing in the Algarve region is identical to that of Portugal (i.e., 51%) [25]. This fact may be related to a higher prevalence of behaviour problems, school failure, and psychiatric morbidity in boys [26,27]. 

Negative correlations were found between children age and QoL, physical well-being, and autonomy and family. A positive relation was also reported between children age and psychological well-being. These results can reveal some influence from stress, school, and developmental demands in children’s domains.

We observed that the parents of both groups had a higher average number of children than their cohort population. However, it is noteworthy that it is higher in the IG than in the CG because it is often mentioned in the literature that primiparas (i.e., first-time or single-child parents) feel the greatest need for advice and have more doubts about the education and development of their children [28]. On the contrary, our data suggest that the experience of past difficulties increases the demand for information.

As for the remaining sociodemographic data, the two groups did not present significant differences in the average age of participants, family structure, gender, and children’s average age. As a result, as mentioned, we can conclude that the groups are comparable in all aspects analysed except for the socio-educational level, which is the reason we took special care to control this variable in our analyses.

A longer use of the programme and access to more information and services is positively related to its perceived usefulness but not to its intensity of use. These facts suggest that the JAF programme is more useful for punctual access where difficulties arise or if specific information is sought, than for regularly seeking new information and content.

Child well-being as perceived by parents in both groups was high and at levels similar to other studies developed in the same region [29]. Physical and psychological well-being was superior to that of peers and social support. Some studies have indicated that parents have a better ability to assess physical aspects rather than emotional and social aspects of their children’s QoL health [19], so it may be important in the future to complement their assessment with their children’s perceptions.

There was no significant difference between children’s QoL for parents participating in the programme and those belonging to the control group. The differences found in QoL dimensions were due to interaction with mothers’ level of education. Mothers with higher education reported greater physical well-being, peers and social support, and school environments of their children as compared to those who had less education. Education level and social class have been reported as the main determinants of quality of life, health, and, in general, of an individual’s control over the environment [30]. Like other online programmes [21], the JAF appears to be less accessible and have less impact on parents with lower educational levels, suggesting that its language and content should be adapted to people with lower socio-educational levels [21,23].

Although it was expected that the JAF would have an impact on children’s QoL, these effects were not found, which raises some reflections. First, it is necessary to consider that QoL is a construct with multiple and powerful structural determinants, which can hardly be modified by a merely informative intervention that can only improve knowledge. Second, the programme fails to penetrate the social class ‘glass ceiling’. The data show that the group in which the information provided by the programme could make a big difference, that is, the one with less education and socioeconomic status, is the one that accesses the programme the least. Given this vulnerability, it would be important to carry out a situational diagnosis [31]. Third, the information provided by the programme that broadens and raises horizons can make parents more aware of their children’s problems and reduce, rather than increase, their perceptions of their quality of life. This paradoxical effect has been observed in other studies conducted on parental effectiveness perceived in families at psychosocial risk [32]. A fourth factor could be constituted by adverse self-selection, that is, by the possible greater adherence to the programme by parents of children with problems. However, the high perceived quality of life suggests that this bias did not occur in this study.

Although there are no differences in quality of life between the IG and CG, we observed positive correlations between various dimensions of QoL, the programme’s use, and perceived usefulness.

The main limitations of this study are related to its observational and cross-sectional design and the choice of quality of life as the only measure to assess the impact of the programme. Though the QoL survey used is a valid, adapt, and robust instrument (i.e., KIDSCREEN), it is important to be aware that this is not an easy construct to measure, since it comprises several aspects of a person’s well-being (i.e., physical, psychological, and social) [19].

In the future, it would be desirable to proceed with an experimental and longitudinal study to establish cause–effect relationships and to be able to determine the impact of the programme over time on several parental factors, such as knowledge, expectations and perceptions about development; children’s education; and sense of parental effectiveness. We believe that an informative programme such as JAF will mainly have an influence on these types of variables. Moreover, in the future, the program should make an effort to involve parents with a lower level of education. These are likely to be the ones who most need and will benefit from this parenting support.

On the other hand, it would be interesting to complement with the biographical narrative interpretative research qualitative study to identify some consequences of the programme not identified in studies of a quantitative nature as well as to explore the reasons the JAF does not seem to ‘reach’ the most disadvantaged groups and parents [24,31]. Furthermore, future studies should assess in detail the impact of different activities and parts of the program, and how these contribute to program outcomes. Finally, to increase the programme’s accessibility and impact, we recommend: (1) adapting the type of language and content to parents of all educational levels; (2) increasing the visibility of videos on the page; (3) developing some content aimed at parents and the challenges of parenthood; and (4) introducing some periodically renewable content.

## Figures and Tables

**Table 1:** Participants’ sociodemographic characteristics.

	Control Group (*n* = 221)	Intervention Group (*n* = 142)	*F*	*p*	*ɳ* ^2^
	*M (SD)*	*M (SD)*			
Parents’ age	39.44 (5.71)	39.71 (4.89)	0.22	0.640	0.00
Children’ age	9.52 (1.46)	9.64 (1.17)	0.66	0.410	0.00
Number of children	1.76 (0.83)	2.18 (0.68)	24.91	0.000	0.06
	% (*f*)	% (*f*)	*χ* ^2^	*p*	*V*
Mothers	96 (221)	97 (138)	0.68	0.410	0.04
Biparental/Single parent	81 (179)/18 (40)	81 (115)/17 (24)	0.06	0.810	0.01
Children—boy	57 (126)	55 (78)	0.19	0.661	0.02
Withholding	89 (75)	95 (135)	2.69	0.101	0.11
Health problems	8 (7)	4 (5)	2.43	0.119	0.10
Secondary/university education	22 (49)/41 (91)	34 (48)/53 (75)	41.99	0.000	0.34
High/Medium/low qualification	26 (51)/42 (83)/32 (63)	54 (71)/42 (55)/4 (5)	47.06	0.000	0.38

Note. *M* = Mean, *SD* = Standard Deviation, *F* = Anova statistic; *p* = *p*-value; *ɳ*^2^ = Eta squared effect size; % = percentage; *f* = frequency; *χ*^2^ = Chi-Square; *V* = Cramer’s V effect size.

**Table 2:** Descriptive and correlation matrix of process outcomes of the JAF programme in the intervention group (*n* = 142).

	*M*	*SD*	Min.-Max.	1	2	3	4
Use of information or services	2.15	0.70	1–4	-	0.32 ***	0.25 ***	0.49 ***
2. How long have you been using the program (years)	7.32	3.73	0–14		-	0.20 *	0.38 ***
3. Frequency of use of the program	1.74	1.03	1–5			-	0.02
4. Program usefulness	2.27	0.57	1–3				-

Note. Frequency of use (1 = occasionally, 2 = monthly, 3 = biweekly, 4 = weekly; 5 = daily). * *p* < 0.05; *** *p* < 0.001.

**Table 3:** Descriptive and correlation matrix of QoL dimensions (*n* = 363).

QoL Dimensions	*M*	*SD*	1	2	3	4	5
Physical Well-being	4.22	0.75	-	0.56 ***	0.39 ***	0.30 ***	0.29 ***
2. Psychological Well-being	4.30	0.49		-	0.45 ***	0.32 ***	0.39 ***
3. Autonomy and Family	3.82	0.66			-	0.60 ***	0.42 ***
4. Peers and Social Support	3.76	0.73				-	0.43 ***
5. School Environment	3.88	0.82					-

Note. Subscales range = 1–5, *** *p* < 0.001.

**Table 4:** Descriptive of QoL in control and intervention group.

Qol Dimensions	Control Group (*n* = 221)		Intervention Group (*n* = 142)	
	*M*	*SD*	*M*	*SD*
Physical Well-being	4.18	0.67	4.31	0.65
Psychological Well-being	4.42	0.49	4.36	0.43
Autonomy and Family	3.99	0.61	3.90	0.58
Peers and Social Support	3.85	0.70	3.84	0.65
School Environment	3.99	0.70	3.95	0.72
Total QoL	4.09	0.46	4.07	0.45

**Table 5:** Differences in QoL between control and intervention groups (*N*_CG_ = 221; *N*_IG_ = 132).

	*F*	*ɳ* ^2^_partial_
Control variables		
Parents’ educational level	4.42 **	0.06
Group	2.15	0.03
Physical Well-being	1.14	0.00
Psychological Well-being	1.28	0.00
Autonomy and Family	3.37	0.01
Peers and Social Support	0.67	0.00
School Environment	3.25	0.01

Note. *F* = Anova Statistic; *p* = *p*-value; *ɳ*^2^_partial_ = Partial Eta Squared effect size. ** *p* < 0.01.

**Table 6:** Correlations between the children’ characteristics, JAF outcomes and QoL (*n* = 142).

	QoL	PhW	PsW	AF	PSS	SE
Children gender	−0.04	0.03	0.06	−0.09	−0.06	−0.18 *
Children age	−0.22 *	−0.27 **	0.23 **	−0.18 *	−0.11	−0.11
School withholding	−0.05	0.03	0.00	−0.10	−0.06	−0.22 *
Health problems	−0.00	0.06	−0.00	−0.06	0.01	−0.05
Siblings	0.05	0.08	0.10	0.03	0.05	0.10
Use of information or services	0.22 *	0.12	0.19 *	0.20 *	0.19 *	0.15
How long have you been using the program	0.10	0.05	0.05	0.12	0.05	0.11
Frequency of use of the program	−0.11	−0.13	−0.08	−0.05	−0.06	−0.09
Program usefulness	0.22 **	0.18 *	0.14	0.22 **	0.16	0.15

Note. QoL—Total Quality of Life, PhW—Physical Well-being, PsW—Psychological Well-being, AF—Autonomy and Family, PSS—Peers and Social Support, SE—School Environment. Children gender (0 = Female; 1 = Male); School withholding, Health problems and Siblings (0 = No, 1 = Yes). * *p* < 0.05; ** *p* < 0.01.

## Data Availability

Data can be available to consultation when request to the corresponding author and with permission of the participants of the study.
